# Diversity of *Crenosoma* species in mustelids with the first molecular characterization of *C. melesi* and *C. petrowi*

**DOI:** 10.3389/fvets.2023.1094554

**Published:** 2023-04-17

**Authors:** Georgiana Deak, Angela Monica Ionică, Calin Mircea Gherman, Andrei Daniel Mihalca

**Affiliations:** ^1^Department of Parasitology and Parasitic Diseases, University of Agricultural Sciences and Veterinary Medicine of Cluj-Napoca, Cluj-Napoca, Romania; ^2^Molecular Biology and Veterinary Parasitology Unit (CDS 9), “Regele Mihai I al Romaniei” Life Science Institute, University of Agricultural Sciences and Veterinary Medicine of Cluj-Napoca, Cluj-Napoca, Romania; ^3^Clinical Hospital of Infectious Diseases of Cluj-Napoca, Cluj-Napoca, Romania

**Keywords:** animal-host association, *Crenosoma* spp., *Meles meles*, mustelids, Romania

## Abstract

Species of genus *Crenosoma* have a wide distribution and are reported in Europe, the Americas, and Asia. Currently, the genus includes 14 nominal species, out of which 9 are parasitic in mustelids. Two species are mostly reported in mustelids from Europe, namely *C. melesi* and *C. petrowi*. Up to now, no genetic sequences are deposited in GenBank for any of the two. The aims of this study were to investigate the distribution, prevalence, and diversity of *Crenosoma* spp. infecting mustelids in Romania and to genetically characterize the species. Mustelids (*n* = 247) were collected over a period of 7 years from different locations in Romania and the respiratory tract was removed and examined for nematodes. Detected nematodes were morphologically identified and fragments of two genes were sequenced. Sampled mustelids included Eurasian badger, *Meles meles* (*n* = 102), Eurasian otter, *Lutra lutra* (*n* = 20), beech marten, *Martes foina* (*n* = 36), European pine marten, *Martes martes* (*n* = 5), steppe polecat, *Mustela eversmanii* (*n* = 1), European mink, *Mustela lutreola* (*n* = 1), least weasel, *Mustela nivalis* (*n* = 2), European polecat*, Mustela putorius* (*n* = 78), and marbled polecat, *Vormela peregusna* (*n* = 1). Nematodes from Eurasian badgers were morphologically identified as *C. melesi* (*n* = 13, 12.74%) and *C. petrowi* (*n* = 3, 2.94%). Nematodes from the beech martens were identified as *C. petrowi* (*n* = 6, 16.66%), *C. vulpis* (*n* = 1, 2.78%) and *Crenosoma* spp. (*n* = 3, 8.33%). Co-infections with two *Crenosoma* species were detected in one beech marten (*C. petrowi* + *C. vulpis, n* = 1, 2.77%) and in one European pine marten [*C. petrowi* + *C. vulpis* (*n* = 1, 20%)]. Two genes of *Crenosoma melesi* and *C. petrowi* were partly sequenced for the first time. We report new host-parasite associations for *M. martes* and *C. vulpis*. However, further studies are needed in order to determine the host-parasite associations and to improve the understanding of the epidemiology of *Crenosoma* nematodes.

## Introduction

Nematodes of the family Crenosomatidae are found in the respiratory tract and sinuses of various mammals (1). The family includes five genera: *Paracrenosoma* Yun and Kontrimavichus, 1936 (in the respiratory system of insectivores), *Troglostrongylus* Vevers, 1923 (in the respiratory system of felids), *Prestwoodia* Anderson, 1978 (in the sinuses of opossums of genus *Didelphis*), *Otostrongylus* de Bruyn, 1933 (in the respiratory tract of seals), and *Crenosoma* Molin, 1861 (in the trachea, bronchi, and bronchioles of carnivores and insectivores) (2). *Molinofilaria* Vuylsteke 1956 (in the bronchi and veins of pinnipeds) is considered similar to *Otostrongylus*, but the classification of this genus remains unclear (Anderson, 1978).

The genus *Crenosoma* is morphologically distinguishable by the presence of a striated and folded cuticle (3). Species belonging to the genus are distributed in Europe, the Americas, and Asia (2, 4–8, 10) (Table 1). The latest review of the genus *Crenosoma* lists 14 nominal species (43), out of which 9 are found in mustelids. Out of these, three species are found in the New World (*C. brasiliense* Vieira et al. 2012, *C. goblei* Dougherty, 1945, *C. hermani* Anderson, 1962) and six in the Old World [*C. melesi* Jančev and Genov, 1988, *C. petrowi* Morozov, 1939, *C. schachmatovae* Kontrimavichus, 1969, *C. schulzi* Gagarin, 1958, *C. taiga* Skrjabin and Petrov, 1928, *C. vulpis* (Dujardin, 1844)].

**Table 1:** Species of *Crenosoma* identified in mustelids: hosts and geographical distribution.

**Species**	**Host**	**Country or region**	**References**
*C. brasiliense* Vieira et al. 2012	**Galictis cuja** ^*^	Brazil	(2)
*C. goblei* Dougherty, 1945	Procyon lotor psora^*^Procyon l. lotor	USA	(5)
	***Martes americana***	USA	(11)
	***Lutra canadensis***	USA	(12)
		USA	(13)
	*Procyon l. lotor*	Canada	(6)
	*Procyon l*. *lotor*	USA	(14)
*C. taiga* Skrjabin and Petrow, 1928	**Mustela sibirica** ^*^	Former USSR	(4)
	***M. putorius***	Russia	(15)
	***Martes martes***	Former USSR	(16)
	***Martes foina***		
	***Meles meles***		
	***Mustela nivalis***		
	***Mustela erminea***		
	***Mustela altaica***		
	***Mustela zibelina***		
	***Gulo gulo***		
*C. schachmatovae* Kontrimavichus, 1969	**Mustela erminea** ^*^	Former USSR	(17)
	***Martes foina***	Lithuania	(18, 19)
	***Neovison vison***		
	***Mustela putorius***		
	***Neovison vison***	Lithuania	(19)
	***Mustela putorius***		
*C. hermani* Anderson, 1962	**Neovison vison** ^*^	Canada	(20)
*C. petrowi* Morozov, 1939	**Martes zibellina** ^*^	Russia	(21)
	***Martes martes ruthega***	Russia	(5)
	***Neovison vison***	Former USSR	Zueva and Belyrov, 1965
	***Gulo-gulo***	Karelian Republic	(22)
	***Martes martes***	Former USSR	(23)
	***Martes foina***		
	***Mustela vison***		
	***Mustela erminea***	Kazakhstan	(24)
	***Pekania pennanti***	USA	(6)
	***Taxidea taxus***	Canada	
	***Martes martes***	Former USSR	(25)
	***Martes foina***		
	***Martes zibellina***	Former USSR	
	***Martes foina***	Former USSR USA Canada	(16)
	*Ursus americanus*	Canada	(17)
	***Martes americana***	Canada	(26)
	***Martes foina***	Italy	(27)
	***Martes martes***	Spain	(28)
	*Vulpes vulpes*	Russia	(29)
	*Nyctereutes procyonoides*		
	***Meles meles***		
	*Canis lupus familiaris*		
	***Martes zibellina***	Russia	(30)
	***Neovison vison***	Russia	(31)
	***Martes foina***	Bulgaria	(25)
	*Ursus americanus*	Canada	(32)
	***Martes foina***	Bulgaria	(33)
	***Martes foina***	Romania	Current study
	***Martes martes***		
	***Meles meles***		
*C. melesi* Jancev and Genov, 1988	**Meles meles** ^*^	Bulgaria	(8)
	***Mustela nivalis***	Spain	(9)
	***Meles meles***	Italy	(34)
	***Meles meles***	Spain	(35)
	***Meles meles***	Norway	(36)
	***Mustela putorius***	France	(10)
	***Mustela putorius***	Germany	(37)
	***Neovison vison***	Spain	(38)
	***Meles meles***	Ireland	(39)
	***Meles meles***	Romania	Current study
*C. schulzi* Gagarin, 1958	**Meles meles** ^*^	USSR	(40)
	***Meles meles***	The Republic of Moldova	(41)
	***Meles meles***	Kirghizstan Uzbekistan Karelian Republic	(16)
*C. vulpis* (Dujardin, 1844)	*Ursus arctos*	Former Yugoslavia	(42)
	*Ursus americanus*	Canada	(7)
	*Taxidea taxus*	Former USSR	Reviewed by (43)
	*Canis lupus familiaris*	Chile	(44)
	Many canid hosts	Eurasia North America	(43)
	***Martes zibellina***	Europe	
	***Lutra lutra***	Former USSR	(16)
	***Gulo gulo***	North America	
	***Meles meles***	Germany	(45)
	***Martes foina***	Germany	(46)
	***Meles meles***	Poland	(47)
	***Meles meles***	Italy	(48)
	***Martes foina***	Portugal	(49)
	***Neovison vison***	Denmark	(50)
	***Mustela putorius***		
	***Martes martes***	Romania	Current study
	***Martes foina***	Romania	Current study

Species marked with “^*^” represent the type hosts. Species in bold are members of Mustelidae.

There is a relatively large body of literature, often confusing, with reports of various *Crenosoma* species in mustelids. For instance, Stunženas and Binkiene (43) list *C. petrowi* as a species distributed in Eurasia but among the hosts, they list two American mustelids. Such a wide distribution over two biogeographical regions is often related to a poor species definition and the absence of genetic data, as most reports are based on morphological identifications. The two most common species of *Crenosoma* reported in mustelids in Europe are *C. melesi* and *C. petrowi*. Surprisingly, prior to the present study, no gene sequences were known for any of the two.

In Romania, *Crenosoma vulpis* infection in carnivores was documented only in foxes (51, 52), and *Crenosoma* spp. in bears (53, 54) with limited knowledge regarding the species diversity and distribution range among other carnivores. Wild carnivores are important reservoirs for parasites that can infect domestic animals and humans (55–58). At the same time, badgers are the least studied group in this direction, even though they are reservoirs for *Mycobacterium bovis* (Infantes-Lorenzo et al., 2019). Romania has a remarkable diversity and abundance of mustelids, with nine extant species recorded in the country (59).

Considering the very limited knowledge and the existence of a wide variety of *Crenosoma* spp. parasites in mustelids, the present paper aimed to investigate the distribution, prevalence, and diversity of these species among mustelids in Romania. Additionally, identified species were also characterized by partial sequencing of two genes, and the risk factors related to sex, age, and geographical localization were analyzed simultaneously.

## Materials and methods

### Samples

Between March 2014 and March 2021, 247 carcasses of mustelids were collected by hunters or found as roadkills in different regions of Romania (Supplementary material 1). Details regarding the sex, age, date and locality of collection were recorded and the carcasses were sent to the Department of Parasitology and Parasitic Diseases of the University of Agricultural Sciences and Veterinary Medicine of Cluj-Napoca where they were kept individually in labeled sealed plastic bags at −20°C until examination. The entire respiratory tract of each animal was removed, and the trachea, the large bronchi, and the bronchioles were thoroughly checked for the presence of nematodes under a stereomicroscope. The lungs were immersed in tap water for a few hours and manually compressed to extract the remaining nematodes. The water was then filtered through sieves and scrutinized for parasites. All nematodes were collected using fine entomological tweezers and washed in physiological saline solution. Nematodes were placed in 4% formalin (for further morphological identification) and 70% ethanol (for molecular analysis). When only one specimen was detected, it was placed in 70% ethanol. A coproscopic and larvoscopic examination was not done due to the freezing and decomposition of the carcasses.

### Morphological identification

Each nematode was temporary mounted on a glass slide in mineral oil and identified based on the morphological descriptions (8). Photographs and measurements of the specimens were taken using an optical microscope (Olympus BX61) connected to a digital camera (DP72 with Cell^∧^F imaging software Olympus Corporation, Tokyo, Japan). The following morphometric features were evaluated in males: body length, body width, number of anterior rings, length and maximum width of the esophagus, lengths of spicules, and length and width of gubernaculum. In females, the following morphological features were evaluated: body length, body width, number of anterior rings, length and maximum width of the esophagus, tail length, and egg size. All sizes are given in micrometers (μm).

### Sequencing and phylogenetic analysis

Genomic DNA was isolated from one or more specimens preserved in ethanol, using a commercial kit (Isolate II Genomic DNA Kit, meridian Bioscience, London, UK), according to the manufacturer's instructions. The samples were processed by means of PCR amplification and bidirectional sequencing of three genetic markers, as previously described (Table 2). Only the samples that yielded high quality sequences of all three markers were further analyzed using MEGA X software (63). The pairwise distances were evaluated, and the evolutionary history was inferred by using the Maximum Likelihood method and Hasegawa-Kishino-Yano model (64).

**Table 2:** Primers used for amplification and sequencing of *Crenosoma* specimens.

**Gene**	**Product (bp)**	**Primer sequence**	**References**
*Cytochrome oxidase* subunit 1 (*cox*1)	~700	LCO1490: GGTCAACAAATCATAAAGATATTGG	(60)
		HCO2198: TAAACTTCAGGGTGACCAAAAAATCA	
Large subunit (LSU) rRNA gene	850-950	391 F: AGCGGAGGAAAAGAAACTAA	(61)
		501 R: TCGGAAGGAACCAGCTACTA	
	850-900	537 F: GATCCGTAACTTCGGGAAAAGGAT	(62)
		531 R: CTTCGCAATGATAGGAAGAGCC	

### Statistical analysis and maps

The statistical analysis was performed using EpiInfo 7 software (CDC, USA). The prevalence of infection and its 95% Confidence Interval (95% CI) were established and the differences among various categories (age, sex, bioregions) were evaluated by chi-square test, and considered significant at *p* ≤ 0.05.

The distribution map was generated using ArcMap 10.6.1 software.

## Results

**Figure 1:**
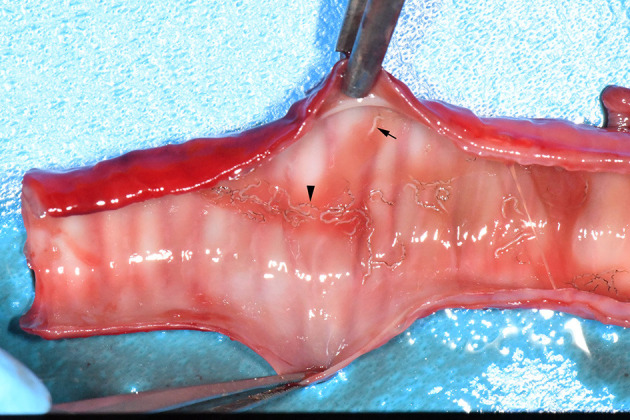
Adult nematodes of *C. petrowi* in the trachea of a badger collected in Borod locality. Arrow head: Female nematodes with a thin, black tube inside; Black arrow: Male nematodes, smaller and white.

The examined mustelids were morphologically identified as Eurasian badger, *Meles meles* (*n* = 102), Eurasian otter, *Lutra lutra* (*n* = 20), beech marten, *Martes foina* (*n* = 36), European pine marten, *Martes martes* (*n* = 5), steppe polecat, *Mustela eversmanii* (*n* = 1), European mink, *Mustela lutreola* (*n* = 1), least weasel, *Mustela nivalis* (*n* = 2), European polecat*, Mustela putorius* (*n* = 78), and marbled polecat, *Vormela peregusna* (*n* = 1). *Crenosoma* spp. nematodes were detected in the trachea, bronchi and bronchioles (Figure 1) of 26 mustelids (10.6%; 95% CI 7.0–15.1), namely: 16 Eurasian badgers (15.7%; 95% CI 9.2–24.2), 9 beech martens (25%; 95% CI 12.1–42.2), and one European pine marten (20%; 95% CI 0.5–71.6). The nematodes collected from the Eurasian badgers were morphologically identified as *C. melesi* (*n* = 13, 12.7%) and *C. petrowi* (*n* = 3, 2.9%). In beech martens *C. petrowi* (*n* = 6, 16.7%), *C. vulpis* (*n* = 1, 2.8%) and *Crenosoma* spp. (*n* = 3, 8.3%) were identified, the latter being in a very bad condition, which rendered them unidentifiable to species level. None of the Eurasian badgers, was co-infected with *C. melesi* and *C. petrowi*. Co-infections with two *Crenosoma* species were detected in one beech marten (CJ007577) [*C. petrowi* + *C. vulpis* (2.8%)] and the only positive European pine marten (CJ007578) [*C. petrowi* + *C. vulpis* (20%)].

**Figure 2:**
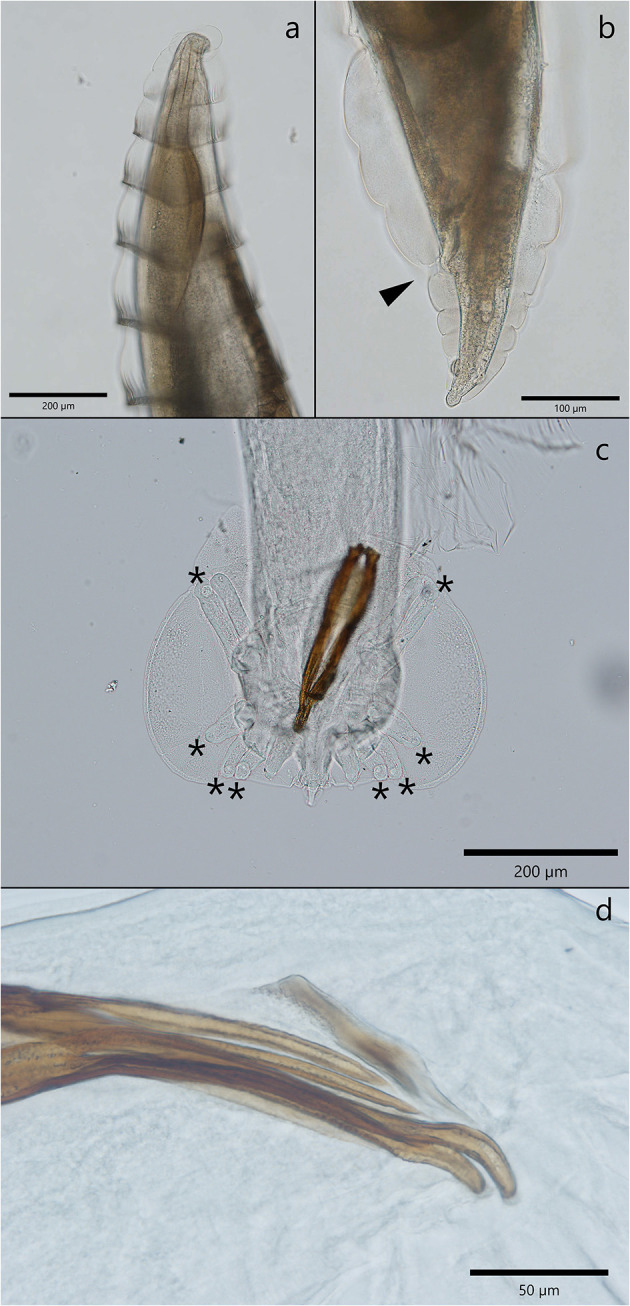
Morphological characteristics of *C. melesi*. **(a)** The specific circular folds. **(b)** Caudal extremity of a female with the cuticular folds visible and the anal opening marked with an arrow head. **(c, d)** Morphological characteristics of a male *C. melesi*. **(c)** The trilobated copulatory bursa with the presence of terminal globular papilla in each ray (asterix); **(d)** note the spicules splited in two branches.

Adult *C. melesi* presented specific circular folds visible along the length of the body (Figure 2a), starting from the middle of the esophagus until after the middle of the body length, with a region in which they were less obvious, followed by their reach back in the region of the anal opening (Figure 2b). The females' body length was between 4.6 and 14.9 mm and their width from 249.9 to 971 μm. From a lateral view, the vulva protruded significantly above the cuticle. Inside the two uteri, eggs in different development stages were observed. The tail has a conic shape with two evident subterminal papillae. The adult males were 4.1 to 5.9 mm in length and 149 to 434.3 μm in width. The cuticular folds were visible until almost half of the body length, after which they disappeared. The copulatory bursa had three lobes, with the lateral ones more developed than the median one. The bursa was sustained by rays which ended in large globular papillae (Figure 2c). The two spicules were almost equal in length, and each was split in two branches in their middle and distal part (Figure 2d).

**Figure 3:**
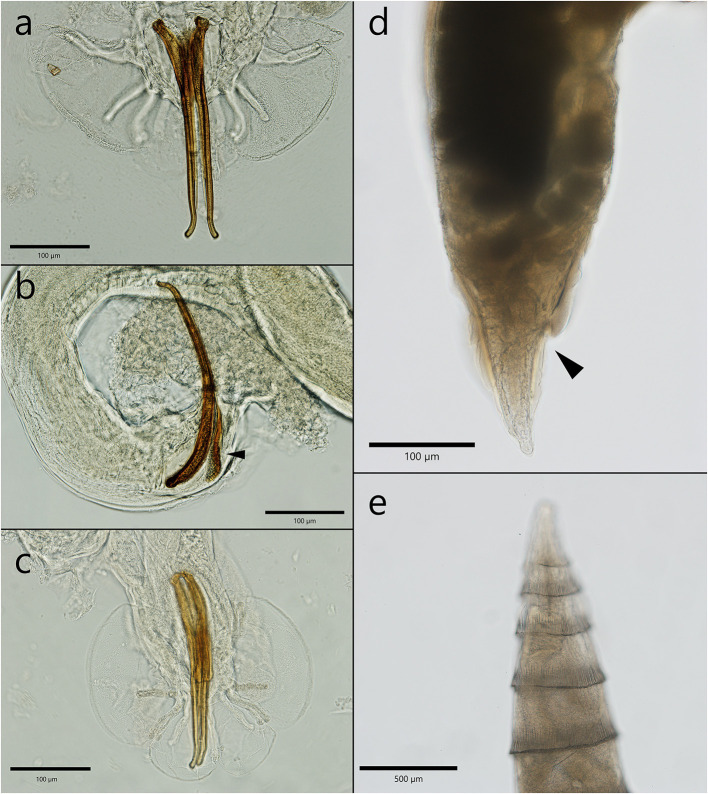
The morphological characteristics of male and female *C. petrowi*. **(a)** Posterior extremity of a male with the two spicules—note the thin protrusion; **(b)** lateral view of a male caudal bursa—the characteristic shape of the gubernaculum (arrow head); **(c)** the posterior extremity of a male with the typical caudal bursa; **(d)** posterior extremity of a female nematode; **(e)** specific folds in the anterior extremity.

*Crenosoma petrowi* adults had a transparent cuticle that formed evident folds that protrude in their posterior part (Figure 3e). The rings formed by the cuticle were visible until half of the body length for female worms and only in the anterior third in males, where the folds stretch and become unapparent. The females detected in Eurasian badger hosts were 6.7–9.2 mm in length and in 402.2–406 μm in width. The ones detected in the two *Martes* species were 3.6 to 5.8 mm in length and 283.3–384.1 μm in width. The vulva was lacking the appendage above the cuticle, and it was localized in the anterior third of the worm. Eggs and larvae were visible inside the uteri. The anal opening was very close to the posterior extremity of the nematode (110–210 μm) (Figure 3d). Male nematodes collected from badgers were 3.4–4 mm in length and 220.3–262.3 μm in width. The male specimens collected from both species of martens were slightly smaller, 2.4–3.2 mm in length and 154.8–224.8 μm in width. The copulatory bursa had three well distinctive parts, and it is sustained by rays. The spicules were almost equal in length and slightly curved at their caudal end (Figures 3a, b). On the dorsal side of the spicules there was a thin protrusion visible in the second third of the length (Figures 3a, c). The gubernaculum had the shape of a barque from a lateral view (Figure 3b).

**Figure 4:**
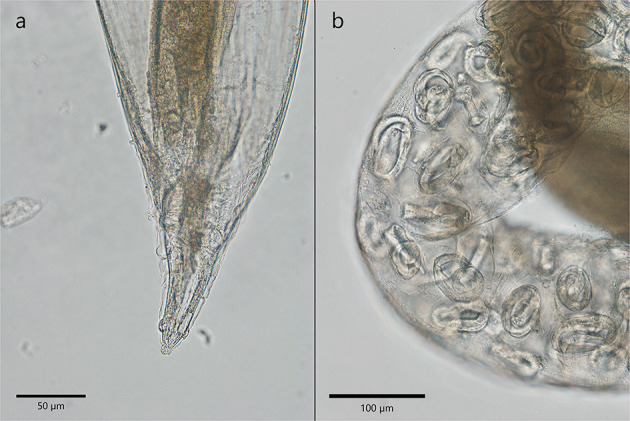
The morphological characteristics of *C. vulpis*. **(a)** The posterior extremity of a female with stretched cuticular folds. Anal opening is visible; **(b)** Note the presence of eggs containing larva in the uterus of a female.

*Crenosoma vulpis* adults have a cuticular sheath that formed evident folds visible in the anterior part and were stretched in the posterior extremity. The females detected in *Martes foina* were 9.4–10.3 mm in length and 348.6–427 μm in width. The ones collected from *Martes martes* were 4.6 to 6.7 mm in length and 357.3–455.4 μm in width. The vulva was positioned almost in the middle of the body, closer to the posterior extremity and the anus was at 117.3–189.3 μm from the caudal extremity (Figure 4a). Two papillae (phasmids) are visible on the lateral parts of the tail. The eggs containing a larva were visible and measured only in one specimen collected from *Martes foina* and had a size of 75.2–76 × 39.7–42.7 μm (Figure 4b). No male specimens of *C. vulpis* were found.

The measurements of all the nematodes that were morphologically characterized are available in the Supplementary material 2.

**Figure 5:**
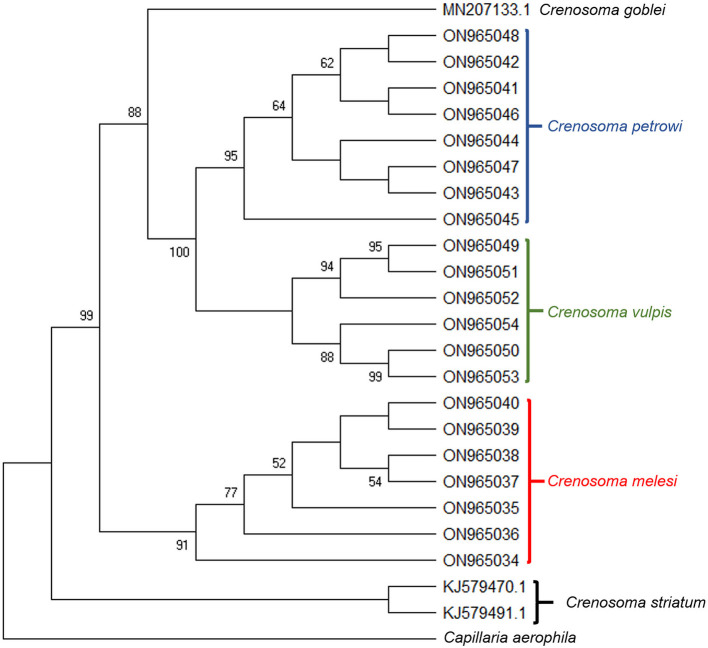
Bootstrap consensus tree inferred from 1000 replicates, taken to represent the evolutionary history of the taxa analyzed. The percentage of replicate trees in which the associated taxa clustered together in the bootstrap test (1,000 replicates) are shown next to the branches. The analysis involved 24 *Crenosoma cox* 1 nucleotide sequences obtained during the present study (21) or retrieved from the GenBank database (3), and one sequence of *Capillaria aerophila*, used as outgroup.

High-quality sequences were obtained for all of the three markers for a total of 21 *Crenosoma* specimens, belonging to 14 hosts: ten Eurasian badgers, three beech martens, and one pine marten. The LSU sequences were highly conserved and insufficient for a clear differentiation among species (Supplementary material 3). However, the differences and distances between the *cox* 1 isolates were in agreement with the morphological identification of the species. The phylogenetic analysis revealed that *C. melesi* formed a separate clade, while *C. petrowi* clustered with *C. vulpis* and is more closely related to *C. goblei* (Figure 5).

According to the bioregion, overall, the differences in the prevalence of infection were significant (*X*^2^ = 13.18; d.f. = 4; *p* = 0.01).

In the Eurasian badgers, juveniles were significantly more frequently infected as compared to adults (*X*^2^ = 8.95; d.f. = 1; *p* = 0.002), while the differences between sex and bioregion were not significant. For the beech martens, no significant differences were identified.

**Figure 6:**
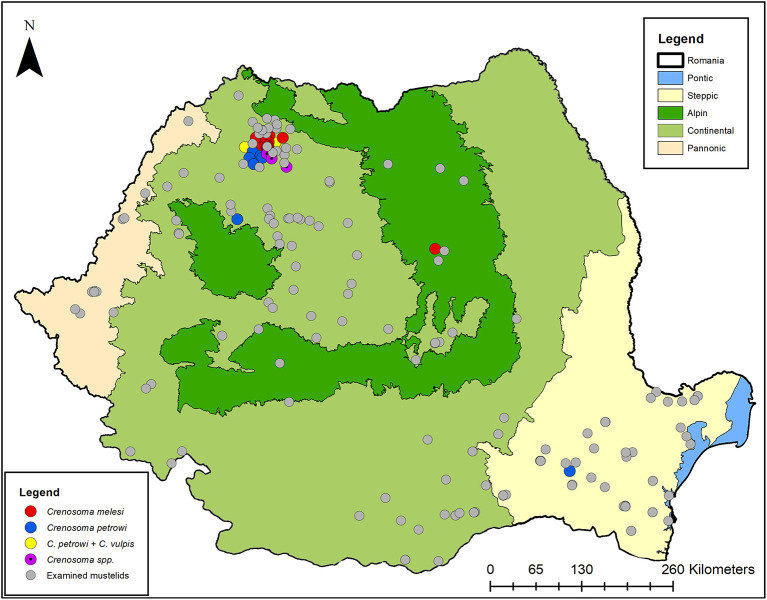
The distribution map of *Crenosoma* spp. in Romanian mustelids.

Thirteen (12.75%) of the Eurasian badgers were infected with *C. melesi*, while the other three (2.94%) harbored *C. petrowi*. Among beech martens, *C. petrowi* was identified in six (16.67%) individuals, of which one (2.78%) was co-infected with *C. vulpis*. The remaining three (8.33%) were positive for *Crenosoma* spp. The statistical data is available in Supplementary material 4. The distribution map of the *Crenosoma* species and the positive hosts is shown in Figure 6.

## Discussion

The genus *Crenosoma* is widely distributed and its species infect a wide variety of mammal hosts. *Crenosoma vulpis*, infecting mainly canid hosts, is the most studied species of the genus, followed by *C. striatum*, parasitic in hedgehogs (Table 1). Although there are numerous studies which report the infection with *Crenosoma*, the identification was based only on the morphological characteristics, correlated with the assumed host specificity. The morphological identification of species is mainly based on the number and aspect of the anterior cuticular folds, the aspect of the female tales and the dimensions and aspects of the copulatory bursa and rays in male nematodes. Nowadays, when genetic tools are largely available, the lack of molecular data could be considered a limitation of the published studies, which could have affected the knowledge on parasite-host associations. Interestingly, up to now, there is very little information about the genetic sequences of *Crenosoma* species. Partial *cox*1 gene sequences are available for only three species in the GenBank (*C. striatum, C. vulpis*, and *C. goblei*), while SSU and/or LSU sequences are known for four species (*C. striatum, C. vulpis, C. goblei*, and *C. mephitidis*).

The present study brings important details regarding the diversity of *Crenosoma* species in mustelids and host-parasite association. Moreover, two species, namely *C. melesi* and *C. petrowi* were reported for the first time in Romania. *Crenosoma melesi* is a respiratory strongyle typically infecting Eurasian badgers, which was initially described in Bulgaria, followed by few reports in other European countries. *Crenosoma melesi* was identified also in other mustelid species (Table 1). Although in our study we found a higher prevalence of infection in juvenile Eurasian badgers, as no data is available from other studies across the range of this host, we cannot conclude that there is an age risk. Generally, higher prevalence in young animals has been documented for other nematode species and it is believed that this could be due to their curious behavior, increased immunity gained with the age, or differences in their food habits (52, 65).

*Crenosoma petrowi* was identified in three mustelid species: *Meles meles, Martes foina*, and *Martes martes*. Additionally, this species is much more similar to *C. vulpis* in regard to its wide range of parasitized animals, including canids and *Ursus americanus*, and also to its wide distribution range (Table 1). Moreover, the present results are in accordance with this statement as based on the phylogenetic analysis, *C. petrowi* is closely related to *C. vulpis* (Supplementary material 3), and genetically distant to *C. melesi*. However, the reports of *C. petrowi* in canid hosts are questionable, as artificial infections of red foxes were unsuccessful (17).

Previously, only *C. vulpis* was morphologically identified in red foxes from Romania (52), and is now morphologically and molecularly confirmed in two mustelid species, *M. martes* and *M. foina*. In red foxes, the abundance of *C. vulpis* has a strong positive relationship with the presence of wetlands, and environmental factors mostly act on the intermediate hosts, regulating the distribution pattern (52). However, animals examined in the present study were grouped based on bioregions, and both animals infected with *C. vulpis* originated in a continental bioregion, located in the same county (Maramureş). This infection could be associated with an endemic area of *C. vulpis* rather than a specific parasite-host association, with mustelids being only accidental hosts.

The absence of *C. vulpis* in Eurasian badgers could be related to a previous misidentification of this species in badgers' hosts, or to a negative parasite-host association. Until more information is available on this topic, we can hypothesize that badgers are unsuitable hosts that may accidentally get infected in endemic areas. The only positive *M. martes* was found to be co-infected with *C. vulpis* and *C. petrowi*, which to the best of our knowledge is the first report of a co-infection with two species of the *Crenosoma* genus in the same host. Similarly, in one *M. foina* we detected the same species association. These findings underline the importance of complementary identification methods, such as molecular typing of specimens, especially for genera that are known to have more than one species parasitic in a particular host. Ideally, morphological identification should be followed by molecular confirmation.

In addition, *C. vulpis* was detected for the first time in a European pine marten. The morphological descriptions of *Crenosoma* specimens from the present study are in accordance with data previously reported (8, 16), with slight differences in morphometrics.

Besides the two species of martens and the badgers, all the other mustelids were negative for infection with *Crenosoma* spp. For *M. nivalis, M. lutreola, M. eversmanii* and *V. peregusna* the lack of infection could be attributed to the low number of examined animals. However, 31 *L. lutra* and 78 *M. putorius* were examined with no *Crenosoma* nematodes detected. Both mustelids are suitable hosts and were previously found to be infected with *Crenosoma* spp. (Petrov, 1940; Kontrimaviciius et al., 1976; Nugaraitė et al., 2014; Kretschmar, 2016). There is only one report of infection with *C. vulpis* in one otter, in the Asian part of the former USSR (16). Most likely, due to its habitat and food preferences consisting mainly in crustaceans and fish (66), this host is not ecologically exposed to infective *Crenosoma* larvae. Infection with *C. taiga* was identified only once in one European polecat from a zoo in Moscow in 1940 (15) and since then, no other reports are available. More recently, infection with *C. schachmatovae* was reported in polecats from Lithuania (18, 19). In the present study, all the examined polecats were negative, and we could speculate that *C. schachmatovae* is absent in Romania (Table 1).

The present paper presents the first complex study on *Crenosoma* parasitic in mustelids and points up the need of respecting specific identification protocols when dealing with multiple-species parasites. Moreover, the study highlights the need for further studies in order to elucidate the host-parasite associations, pathological implications on mustelid hosts, as well as the exploration of other gene sequences and their suitability for phylogenetic taxonomy.

## Conclusions

There are three species of the genus *Crenosoma* infecting Romanian mustelids. Co-infections with two species of the genus in the same animal host are possible. We report a new host-parasite association for *M. martes* and *C. vulpis*. Sequences of *C. melesi* and *C. petrowi* were described for the first time. The *cox1* difference between the identified species is in accordance with the morphological identification. Further studies are needed in order to determine the host-parasite associations and to improve the understanding of the epidemiology of *Crenosoma* nematodes.

## Data availability statement

The datasets presented in this study can be found in online repositories. The names of the repository/repositories and accession number(s) can be found in the article/Supplementary material.

## Ethics statement

The animal study was reviewed and approved by the ethical decision nr. 232 23.11.2020, approved by Bioethics Committee of USAMV Cluj-Napoca.

## Author contributions

GD helped with the collection of the carcasses, performed the necropsies, morphologically identified the nematodes, analyzed the data, and wrote the manuscript. AI performed necropsies, the molecular work, and the statistical analysis. CG performed the necropsies and revised the manuscript. AM coordinated the study, financially supported the molecular work, and critically revised the manuscript. All authors contributed to the article and approved the submitted version.
